# Experimental demonstration of tractor millimeter wave beam propulsion

**DOI:** 10.1038/s41598-025-02791-5

**Published:** 2025-05-20

**Authors:** Masayuki Takahashi, Toshiki Yamada, Ryutaro Minami, Tsuyoshi Kariya, Kohei Shimamura

**Affiliations:** 1 Department of Aerospace Engineering, Tohoku University, Sendai, 980-8579 Japan; 2 Plasma Research Center, University of Tsukuba, Tsukuba, 305-8577 Japan; 3 Department of Aeronautics and Astronautics, Tokyo Metropolitan University, Hino, 191-0065 Japan

**Keywords:** Millimeter-wave beam, Plasma, Beamed energy propulsion, Aerospace engineering, Electrical and electronic engineering

## Abstract

Tractor millimeter-wave beam propulsion (TMiP), which obtains a propulsive force by receiving a high-power tractor beam from the front side of the vehicle, was proposed for space missions such as rocket launches from Earth and planetary takeoff missions. A beam irradiation experiment with a gyrotron device was conducted for a model rocket with a polytetrafluoroethylene (PTFE) lens to collect the tractor beam power, facilitating gas breakdown and plasma generation at the focal point. A high-pressure gas was formed via plasma heating, interacting with the PTFE lens mounted on the front of the vehicle and generating a propulsive force for rocket launching. A parametric study was conducted by changing the pulse width, which showed that the maximum thrust performance was achieved when the plasma front propagating toward the beam source did not protrude from the air-breathing intake at the front of the vehicle. Additionally, computational simulations for electromagnetic wave propagation and compressible fluid dynamics indicated that the thrust performance could be improved by decreasing the rocket diameter due to shock wave concentration.

## Introduction

Recently, a reduction in rocket launch costs has been required to accelerate space development in various fields, such as commercial use, national defense, and academic research. This cost reduction can be achieved if a reusable rocket technology is established, as discussed in SpaceX. However, as a different idea for cost reduction, the beamed-energy propulsion concept, which obtains a propulsive force by irradiating an intense pulse beam from the ground oscillator to the flying rocket, offers another approach to reducing launch costs^1–49^. A microwave rocket is a beamed-energy propulsion technology that generates a high impulse for rocket launches using millimeter-wave beam pulses. After irradiation with the millimeter-wave beam, dense plasma can be generated inside the nozzle via a gas breakdown process. The plasma front propagates toward the beam source, absorbing beam energy and heating during the pulse width. The energy of the millimeter-wave-driven plasma was then transferred to that of the neutral gas, inducing a strong shock wave with a detonation-like structure. The high pressure of the induced shock wave interacts with the thruster wall to generate thrust. Using atmospheric air as fuel to create plasma and shock waves could significantly reduce fuel costs. In addition to reducing fuel, the complex gas-burning engine can be omitted, enhancing rocket reusability and lowering overall space transportation costs.

To establish the microwave rocket concept, the thrust performance was experimentally measured using single and repetitive millimeter-wave beam pulses in previous studies^1–3^. In the single-pulse experiment, a high thrust performance was achieved by inducing millimeter-wave-driven plasma and shock waves and exhausting the plasma downstream of the nozzle; however, the thrust performance under repetitive pulse operation decreased with time^2^. Thrust performance decreased because residual plasma can be generated after the first beam pulse is irradiated into the rocket nozzle, and it absorbs the millimeter-wave-beam energy irradiated from downstream to upstream of the nozzle at the following pulse timing. This residual plasma absorption caused the plasma generation position to shift downstream of the nozzle during the repetitive pulse operation, eventually leaving the nozzle and reducing thrust to zero. Therefore, it is necessary to avoid energy absorption by the residual plasma to maintain a high impulse of the rocket under repetitive pulse operation.

**Fig. 1:**
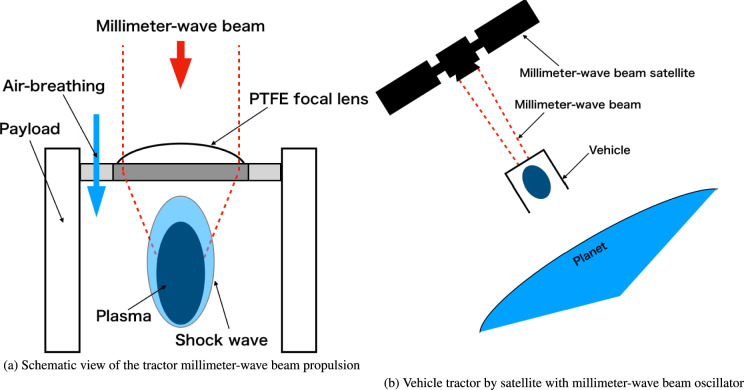
Concept of the tractor millimeter-wave beam propulsion system. Detailed thruster design and its operation by receiving the beam energy from satellites.

In the conventional microwave rockets, plasma exhaust and beam injection occur at the same port in the rocket nozzle; therefore, if these ports could be separated, the effects of the residual plasma could be avoided, maintaining a high thrust performance under repetitive pulses. A tractor millimeter-wave beam propulsion (TMiP) concept is proposed to achieve port separation, avoiding a decrease in performance under repetitive pulses, as shown in Fig. 1a. In the tractor-beam concept, the beam source is established at the front side of the rocket by launching a satellite with a high-power millimeter-wave beam oscillator, as shown in Fig. 1b. The millimeter-wave beam irradiates from the front to the rear of the vehicle, focusing on the rear. In this study, a PTFE lens is mounted at the front of the vehicle to concentrate the incident beam energy. Dense plasma forms at the focal point of the rear side of the vehicle, inducing a strong shock wave. The shock wave is confined inside the vehicle cylinder, and its high pressure can push the vehicle toward the beam source direction (flight direction) while exhausting the plasma toward the rear side of the vehicle. After each pulse, the gas inside the thruster was refreshed by breathing from the intake port mounted on the front of the vehicle. This system separates the beam injection port from the plasma exhaust port during repetitive pulse operations because the next beam pulse is irradiated from the front side of the vehicle. However, the plasma is exhausted to the rear.

Moreover, it is believed that the tractor-millimeter-wave beaming technology is not only effective for rocket launches from Earth but is also suitable for payload collection from planets such as Mars. For instance, future missions could use electric propulsion for manned exploration of Mars. Currently, such missions are one-way, and there is no return method from Mars to Earth because the payload mass to the planet is limited, and the rocket launch system from the planet cannot be loaded. In this scenario, if the satellite with the beam source is previously injected into orbit around the planet, the propulsion force, which is sufficient to launch the spacecraft from the surface of the planet, can be provided to the vehicle from the satellite via the tractor millimeter-wave beam, returning the manned spacecraft to Earth, as shown in Fig. 1b. Additionally, this propulsion concept can be used as a driver for space elevator systems. Given its potential applications, this propulsion concept requires aggressive development to support future space missions.

In addition to the discussion of the aforementioned concepts, an increase in the beam power will be required for the practical application of the launch system in the future, as discussed in previous research. Although the beam sources used in our experiments are small (on the order of several hundred of kW) in comparison to actual launches, the beam power and antenna size for beam transmission should be increased to launch a rocket of practical size for space missions. In previous estimations for launching satellites of 8 kg, the orbital simulation indicated that a beam power of 80 MW and an antenna diameter of 90–175 m are required to inject payloads into a low earth orbit having a 300-km altitude^4^. To achieve such high-power beam irradiation, a clustering system consisting of multiple MW-class gyrotrons is required. For ground-based beam oscillators, the combination of flywheel batteries and phased-array antennas is one of the most promising candidates studied for this transmission system. In addition, when the proposed TMiP system is driven by a tractor beam from space, a large space solar power satellite (SSPS) could serve as a promising beam source. In this case, the beam clustering technology developed for ground-based oscillations can be applied to a high-power beam source in the SSPS.

In the previous studies on TMiP, numerical simulations were conducted to evaluate the beam focus, plasma generation, and thrust performance by combining electromagnetic wave propagation, plasma reaction diffusion, and shock wave propagation modules^20,26^. Simulations indicated that sufficient thrust performance could be achieved using the concept of TMiP; however, an experimental demonstration of plasma and thrust generation has not been conducted for the propulsion concept. Therefore, this study aims to experimentally demonstrate the TMiP and evaluate its thrust performance by irradiating a 28-GHz millimeter-wave pulse beam onto the thruster model. Although a propulsion force can be obtained in the background gas other than air, as for rocket launches from planets such as Mars, the demonstration in this paper used air as the background gas for simplicity.

## Methods

### Thruster model used in experiments

**Fig. 2:**
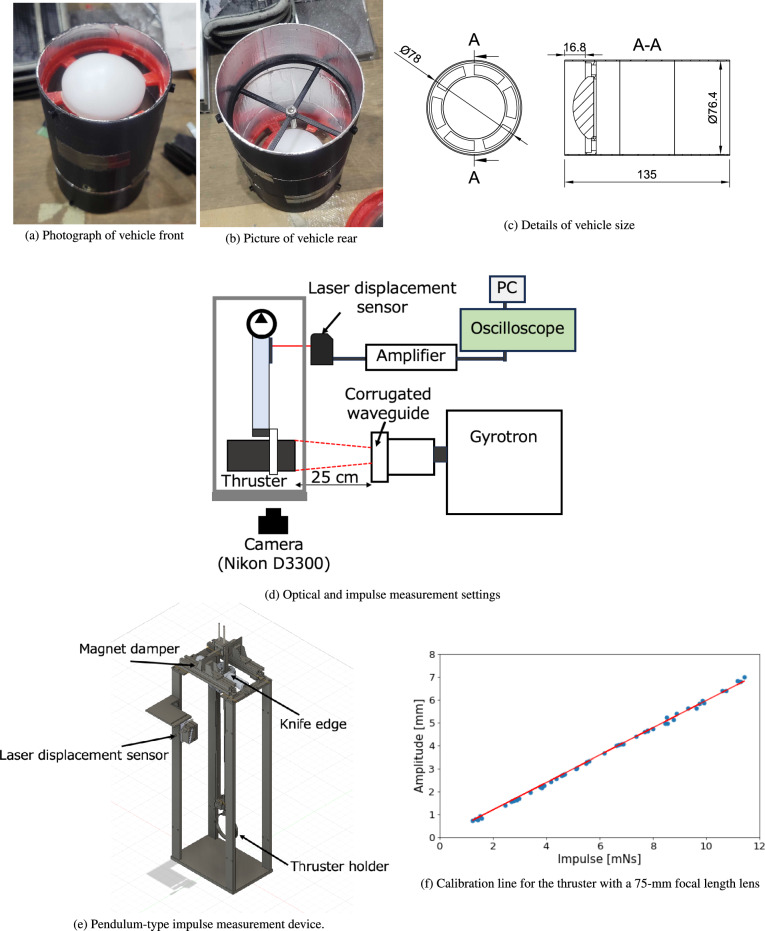
Thruster used in the experiment. Optical setting, impulse measurement device, and calibration line. 3D design of the impulse measurement device was drawn using Autodesk Fusion 360 (version 2.0.18441). This software was downloaded from https://www.autodesk.com/jp/education/edu-software/fusion.

Figure 2a–c illustrates the developed cylindrical thruster. A PTFE lens (LAT075, Thorlabs) with a transmittance of over 90% for GHz beams was mounted on the front side of the vehicle, as shown in Fig. 2a. To be specific, a rough estimation of the PTFE lens transmittance for 28-GHz beams was conducted using the following equations:

1 $$\begin{aligned} T&=\biggl (1-|\Gamma _1|^2 \biggr )^2 \textrm{e}^{-2 \alpha z_w}, \end{aligned}$$

2 $$\begin{aligned} \alpha&= \omega \sqrt{\frac{\mu \epsilon }{2}(\sqrt{1+\textrm{tan}^2\delta }-1)}, \end{aligned}$$

where *T* represents the transmittance, $\Gamma _1=(1-\sqrt{\epsilon _r})/(1+\sqrt{\epsilon _r})$ is the reflection rate between air and dielectric, $\alpha$ is the damping coefficient in the dielectric, $z_w$ represents the dielectric thickness, $\omega$ represents the angular frequency of the beam, $\mu$ represents the magnetic permeability, $\epsilon$ represents the permittivity, $\epsilon _r$ represents the relative permittivity, and $\textrm{tan}\delta$ represents the loss tangent. For 28-GHz beams and the PTFE lens, which has $z_w=1$ cm, $\epsilon _r=2.1$, and $\textrm{tan}\delta =2.0\times 10^{-4}$, the transmittance evaluated by the above equations is 96%^50^. A holder fixes the lenses. An air intake with a width of 8 mm was placed on the lens holder to refresh the air inside the main body of the cylinder. Additionally, as an ignition pin, an M4 bolt was mounted at the focal point of the PTFE lens to induce air breakdown and generate dense plasma, as shown in Fig. 2b. The cylindrical body, lens holder with air intake, and ignition pin holder were fabricated from PLA resin using a 3D printer (Finder3, FLASHFORGE). Aluminum tape was putted on the cylinder interior to confine the incident beam inside the cylinder and suppress energy loss due to beam divergence. In addition to a LAT075 lens with a focal length of 75 mm, a PTFE lens with a focal length of 91 mm (LAT100, Thorlabs) was used for the parametric studies.

As a thermal design issue on the PTFE lens, when plasma or gas heated by incident beams to several thousand of Kelvins^10^ contacts the PTFE lens, it may be damaged. However, because the interaction interval between this high-temperature plasma and the PTFE lens was limited to a few milliseconds, the total heat flux to the PTFE lens was negligible. Therefore, we examined the surface of the PTFE lens after the experiment and discovered no significant evidence of intense damage, ablation, or destruction. In addition, the toxic and other effects of PTFE evaporation were considered negligible. However, because this damage or evaporation could be enhanced when the beam power is increased in actual rocket launch situations, alternative heat-resistant materials or cooling system may be required for high-power beam irradiation. This topic will be discussed in future studies.

In addition, we would like to discuss thermal damages on the ignition pin holder. Plasma-front propagation occurred from the tip of the ignition pin and the plasma heated by the microwave beam did not directly contact the ignition pin holder. Therefore, our experiment revealed no damages to the pin holder. Even if the beam power is increased for practical use in future launch missions, serious damage to the ignition pin holder can be avoided because it does not lie along the plasma-front propagation axis.

#### Optics and impulse measurement settings in experiments

A 28-GHz gyrotron with a maximum power of 500 kW, which was used at the University of Tsukuba, was irradiated onto the vehicle to induce plasma and shock waves, as shown in Fig. 2d. In this study, single-pulse beams with powers of 100, 130, and 210 kW were used. Moreover, the pulse width of the beam was changed in a range from 0.6 to 2.5 ms. During the pulse width, a roughly constant beam power was maintained in the gyrotron device. The beam was outputted from a gyrotron device and transmitted via a corrugated waveguide. The inner diameter of the corrugated waveguide was 63.5 mm, and the distance between the waveguide exit and thruster was 25 cm. The millimeter-wave beam diverged during this beam transmission in air, following a Gaussian beam profile. The beam power densities at the exit of the corrugated waveguide were 0.077–0.16 $\hbox {GW/m}^2$ for the beam powers of 100–210 kW.

The plasma images induced by the thruster were captured using a digital camera (D3300, Nikon) at a resolution of 6,000$\times$4,000 pixels. The exposure time of the camera was set to 4 s, and integral images were acquired from the beginning to the end of the discharge process. The plasma inside the thruster was visualized by building the main body of the cylinder with an observation window (a hollow window); however, this window was closed to confine the shock wave inside the thruster when the thrust performance was measured. During the visualization, the vehicle was fixed, and no thrust measurement device was used. Plasma samples were collected from the side and front of the vehicle.

A pendulum-type impulse-measurement device was used to evaluate the thrust performance of the vehicle, as shown in Fig. 2e. This device has a pendulum rotating around a knife edge and a magnetic damper to suppress noise and accelerate the static stopping of the pendulum. The displacement of the pendulum was measured using a laser displacement sensor, which consisted of a sensor head (IL-S100, Keyence), an amplifier unit (IL-1000, Keyence), and a DC power supply (KZ-U3, Keyence). The laser displacement sensor was connected to a digital oscilloscope (Analog Discovery2, DIGILENT) to obtain the output signal. Based on the equation of motion of the pendulum, the maximum amplitude of the oscillation can be converted into an impulse calculated as follows:

3 $$\begin{aligned} A_\textrm{max}=\frac{I l_1 l_2}{\sqrt{aM_1-bM_2}gJ}, \end{aligned}$$

where $A_\textrm{max}$ is the maximum amplitude of the oscillation, *I* is the impulse applied to the vehicle, $l_1$ is the distance between the thrust generation position and the fulcrum, $l_2$ is the distance between the displacement measurement position and the fulcrum, *a* is the distance between the center of gravity of the lower arm, *b* is the distance between the center of gravity of the upper arm, $M_1$ is the mass of the lower arm, $M_2$ is the mass of the upper arm, *g* is the gravitational acceleration, and *J* is the pendulum moment of inertia. The impulse measurements were repeated five times, and the average value was used as the measured value. Additionally, the standard deviation of the impulse is plotted as an error bar in the thrust measurement. The calibration line of this measurement device was evaluated by applying a known impulse from an impulse hammer with a load cell (LMA-A-5N, Kyowa) to the pendulum. Figure 2f shows the calibration line for the vehicle with a 75-mm focal length lens, which was used to evaluate the impulse obtained by the millimeter-wave beam irradiation. The calibration line was remeasured when the vehicle model was changed.

#### Evaluation of momentum coupling coefficient

The momentum coupling coefficient, which represents the thrust efficiency per unit of beam power, was calculated using the following formula:

4 $$\begin{aligned} C_m=\frac{I}{P \tau }, \end{aligned}$$

where *I* is the impulse, *P* is the beam power, and $\tau$ is the pulse width of the incident beam. To consider the beam divergence from the beam irradiation port to the front of the vehicle, the beam power at the front of the vehicle was calculated as

5 $$\begin{aligned} P=P_0\biggl [1-\textrm{exp}\biggl (\frac{-2R_{th}^2}{w(z)}\biggr )\biggr ], \end{aligned}$$

where $P_0$ is the gyrotron output power measured using a dummy load, $R_{th}$ is the thruster radius, and *w*(*z*) is the beam spot radius at a distance *z* from the beam irradiation port. Based on the theoretical prediction of Gaussian beam divergence, *w*(*z*) at a distance *z* from the waveguide exit was calculated as

6 $$\begin{aligned} w(z)=w_0\sqrt{1+\biggl (\frac{\lambda z}{\pi w_0^2} \biggr )}, \end{aligned}$$

where $w_0$ is the beam waist radius, and $\lambda$ is the beam wavelength. In this experiment, $w_0$ was 20.3 mm, *z* was 250 mm, and $R_{th}$ was 38 mm, resulting in

7 $$\begin{aligned} P=0.735 P_0. \end{aligned}$$

This *P* estimated at the lens entrance was used for the evaluation of $C_m$.

#### Simulation methods and conditions for FDTD model

The beam power density is numerically obtained by solving 3D Maxwell’s equations using the FDTD method as follows:

8 $$\begin{aligned}&\frac{\partial \textbf{E}}{\partial t} = \frac{1}{\epsilon } \nabla \times \textbf{H}, \end{aligned}$$

9 $$\begin{aligned}&\frac{\partial \textbf{H}}{\partial t} = -\frac{1}{\mu }\nabla \times \textbf{E}, \end{aligned}$$

where $\textbf{E}$ is the electric field vector, $\textbf{H}$ is the magnetic field vector, $\epsilon$ is the permittivity, and $\mu$ is the magnetic permeability, respectively. The electromagnetic-wave source used the total-field scattered-field formulation to avoid nonphysical reflection at the source position. In Eq. (9), $\mu$ was set to the value in vacuum. $\epsilon$ was calculated as $\epsilon _r \epsilon _0$ in the position of the PTFE lens and $\epsilon _0$ in the other areas, where $\epsilon _r$ was the relative permittivity, and $\epsilon _0$ was the permittivity in vacuum, respectively. $\epsilon _r=2.1$ was employed for the PTFE lens, which corresponds to the real part of the complex permittivity of PTFE. Because PTFE has a high transmission efficiency for GHz beams, the imaginary part of the complex permittivity, which plays the role of beam energy absorption, was not considered in the simulation. However, the refraction effect of the incident beams can be reproduced by introducing the real part of the permittivity.

The 3D simulation domain had lengths of 100 mm, 100 mm, and 150 mm in the *x*-, *y*-, and *z*-directions, respectively. Gaussian beam irradiation with a beam power of 210 kW and frequency of 28 GHz was numerically reproduced by setting the beam source in the vicinity of $z=0$ mm, and its propagation direction was toward the *z*-direction. To determine the spatial distribution of the electric field intensity in the beam source, it is assumed that the waveguide exit with a beam waist radius of 20.3 mm was set at $z=-250$ mm. The electric field distribution at $z=0$ mm is calculated using Eq. (6) while considering the Gaussian beam divergence from this gyrotron exit, which was used as the beam source at $z=0$ mm. The 3D domain had computational grids whose interval of $\lambda /15$, where $\lambda$ was the 28-GHz beam wavelength. For the outer boundaries, a 2nd-order Mur absorbing boundary was used to reproduce the open boundary condition^51^. The PTFE lens with a curvature radius of 32.25 mm and a thickness of 16.9 mm was set at $(x,~y,~z)=(50,~50,~5)$ mm, reproducing the experiment for the $F=75$ mm lens.

#### Simulation methods and conditions for CFD model

Compressible fluid dynamics during millimeter-wave heating were numerically reproduced by solving the 2D axisymmetric Euler equation as follows:

10 $$\begin{aligned} \frac{\partial \textbf{Q}_\textrm{f}}{\partial t}+\frac{\partial \textbf{E}_\textrm{f}}{\partial z}+\frac{\partial \textbf{F}_\textrm{f}}{\partial r}+\textbf{H}_\textrm{f}=\textbf{S}_\textrm{f}, \end{aligned}$$

where $\textbf{Q}_\textrm{f}$ is the conservative variables vector; $\textbf{E}_\textrm{f}$ and $\textbf{F}_\textrm{f}$ are the inviscid flux vectors in the *z*- and *r*-directions; *z* and *r* are the axes in the axisymmetric system; *t* is time; $\textbf{H}_\textrm{f}$ is the axisymmetric term vector; and $\textbf{S}_\textrm{f}$ is the heating source vector. The cell-centered finite volume method was used for spatial discretization. The AUSM-DV method^52^ was employed to evaluate the inviscid flux vectors using a third-order MUSCL method^53^. In the MUSCL method, a minmod limiter was utilized to fulfill the total variation diminishing condition. Time integration was performed using the second-order Runge-Kutta method.

The heat-source term vector $\textbf{S}_\textrm{f}$ in Eq. (10) was calculated as $\textbf{S}_\textrm{f}=[0,~0,~0,~\mu _b S_0/ \lambda ]^\textrm{T}$, where ${\mu _b}$ was the energy conversion rate from the plasma to the neutral fluid, which was set to 30% in this study^17,54^, and $S_0$ was the beam power density. The heating source with an area of ${\lambda }\times {\lambda }$ was placed inside the thruster, which was moved toward the upstream at a speed of $U_i$, where $U_i$ was the propagation speed of the heating region. To model this heating region in CFD, 3D FDTD simulations evaluated the 1D spatial profile of the Poynting vector $\textbf{S}_\textrm{p}(x,~y,~z,~t)=\textbf{E}\times \textbf{H}$ along the *z*-axis. This time-varying pointing vector was averaged over one period of the incident electromagnetic wave, which was used as $S_0$ in the CFD simulation as follows:

11 $$\begin{aligned} S_0(z)=\frac{1}{T_\textrm{b}}\int _0^{T_\textrm{b}}|\textbf{S}_\textrm{p}(0,~0,~z,~t)|~\textrm{d}t, \end{aligned}$$

where $T_\textrm{b}$ is the period of the incident electromagnetic wave, and $S_0(z)$ is a function of *z*. Additionally, $U_i$ of the heating area in the CFD was evaluated by linear interpolation of the experimental data^10^, which shows the relationship between the beam power density and the propagation speed of the plasma front. Because $S_0(z)$ depends on the *z*-position, $U_i(z)$ also changes depending on *z*, which was considered in the movement of the heating region in the CFD simulations.

A computational grid was constructed using the multi-block method to simplify the boundary condition-setting process. The simulation domain was sufficiently large to suppress nonphysical reflection from the outer boundaries. A zeroth-order extrapolation was used for the outer boundary, and an inviscid reflection boundary condition was applied to the thruster wall. A grid with a uniform interval of 0.8 mm was used inside the thruster; however, for the vehicle outside, the grid interval gradually increased toward the open boundaries. The thruster model simulated by CFD corresponded to that in the experiment with a lens of $F=75$ mm. The inside diameter of the vehicle ($\Phi$) was set to 76 mm, which was the same as the experimental model. Additionally, to understand the effect of the air-breathing intake of the vehicle, a thruster model without an intake was compared with that with an intake. For the parametric study, the smaller inside diameter model with $\Phi =25$ mm was simulated, having no intake.

## Results and discussions

### Plasma observation for thruster with a 75-mm focal length lens

**Fig. 3:**
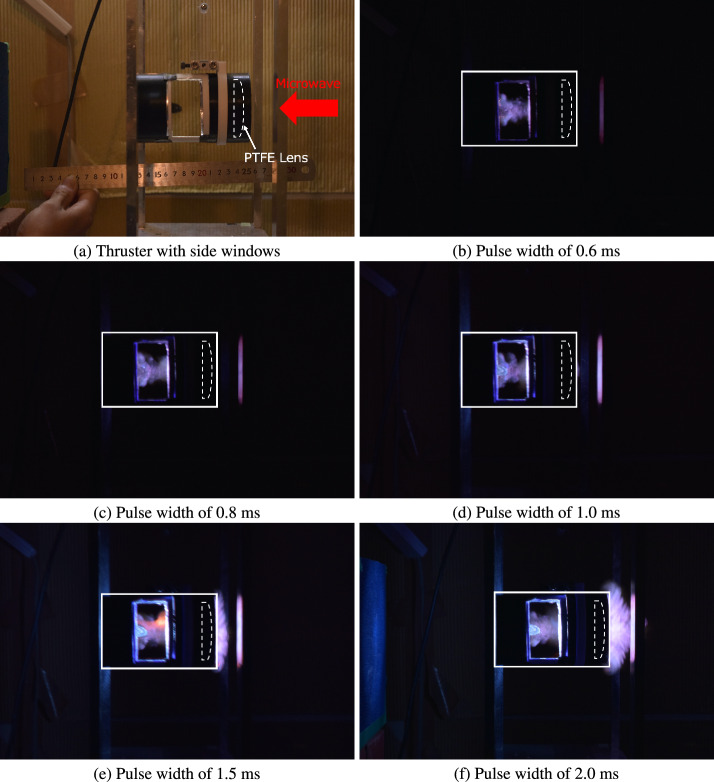
Plasma formations with different pulse widths at 210 kW. The hollow observation window is placed on the thruster, and the integral images are captured using a digital camera. The millimeter-wave beam is irradiated from the right to the left side. The pulse widths of the incident beam are varied in a range of 0.6–2.5 ms.

Figure 3 shows the integral images for the plasma formation at the different pulse widths $\tau$ when the millimeter-wave beam with 210-kW power was irradiated from the front to rear sides of the vehicle. An ignition pin is visible in the observation window shown in Fig. 3a, which is located at the focal point of the PTFE lens mounted on the front of the vehicle. As shown in Fig. 3, millimeter-wave beam-induced plasma formed around the focal point of the lens because a high-electric-field region was formed by focusing by the PTFE lens. As indicated in the previous discharge experiment performed by 28-GHz beams^10^ and previous simulations^27–29^, the ionization process and plasma formation around the high electric field region were sustained by the processes of gas heating, accumulative ionization, and thermal ionization after a rapid increase in the electron temperature via Joule heating. The plasma front propagates from the focal point to the beam source direction (front side of the vehicle) while absorbing the subsequent beam energy at the plasma front. The high electron temperature was maintained by beam energy absorption, which induced an increase in the neutral gas temperature through the vibrational-translational relaxation and generated a shock wave inside the vehicle cylinder.

**Fig. 4:**
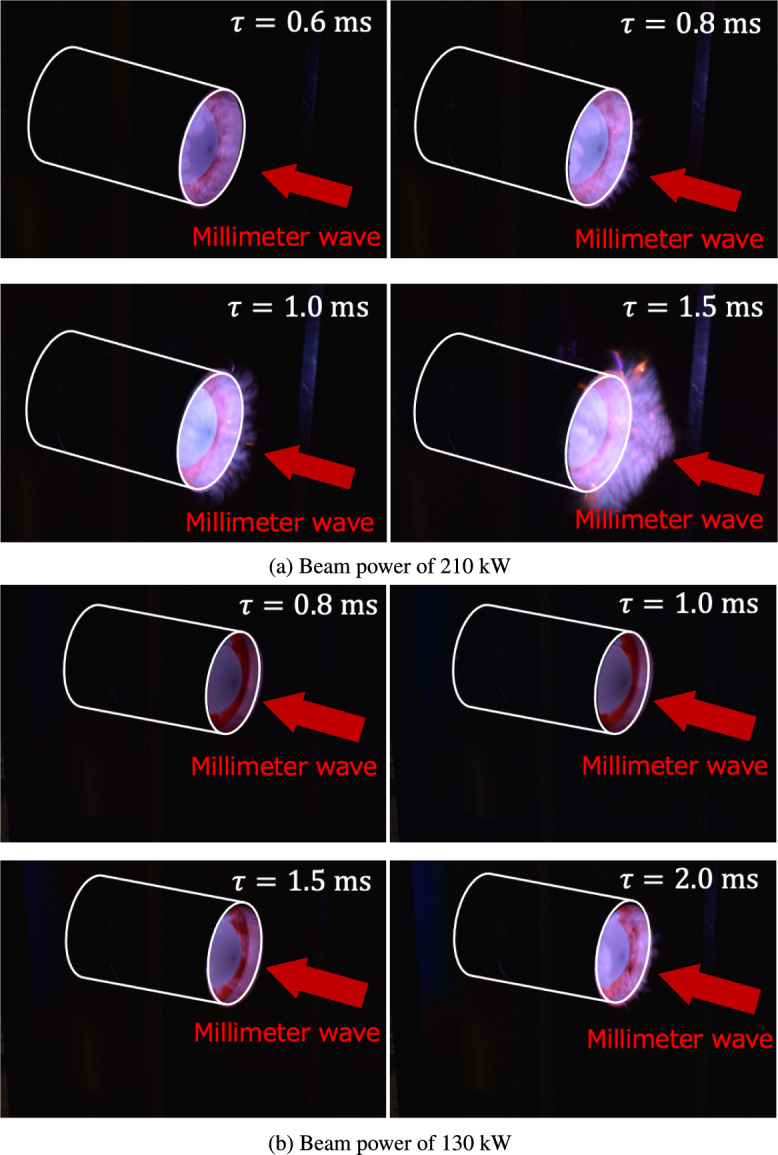
Front side view of plasma-front propagation. The observation window is removed from the thruster, and the red arrow indicates the beam irradiation line. The integral images are obtained using a digital camera. The beam powers and pulse widths $\tau$ are changed.

As indicated in Fig. 3, the plasma front propagation distance can change depending on the pulse width, which may affect the thrust performance of TMiP. At $\tau \le$ 0.8 ms (Fig. 3b and c), the plasma front remained within the cylinder of the vehicle, demonstrating that the concept, which generates plasma and shock waves inside the cylinder of the vehicle to obtain high thrust performance, was successfully achieved. However, at $\tau \ge$ 1 ms, the light emission from the plasma was observed at the right side of the vehicle front, in addition to the observation window, revealing that the plasma front protruded from the end of the cylinder of the vehicle through an air-breathing port, as shown in Fig. 3d–f. This plasma-front outflow at pulse widths of 1–2 ms was more clearly observed in the front side view of the vehicle for the 210-kW beams, as shown in Fig. 4a, which could decrease the thrust-to-power efficiency due to energy loss from the cylinder of the vehicle. Here, focusing on the front view of the vehicle at $\tau =1.5$ ms (Fig. 4a), the formation of branching plasma structures was captured at the front of the vehicle, which could correspond to the structure of “diffusively branched plasmoids” observed in the past discharge experiment^10^. It is considered that this branched plasmoid structure is formed due to interactions between the electromagnetic wave, plasma, neutral gas dynamics, and chemical reaction processes; however, a detailed mechanism of pattern formation will be discussed in a forthcoming paper.

**Fig. 5:**
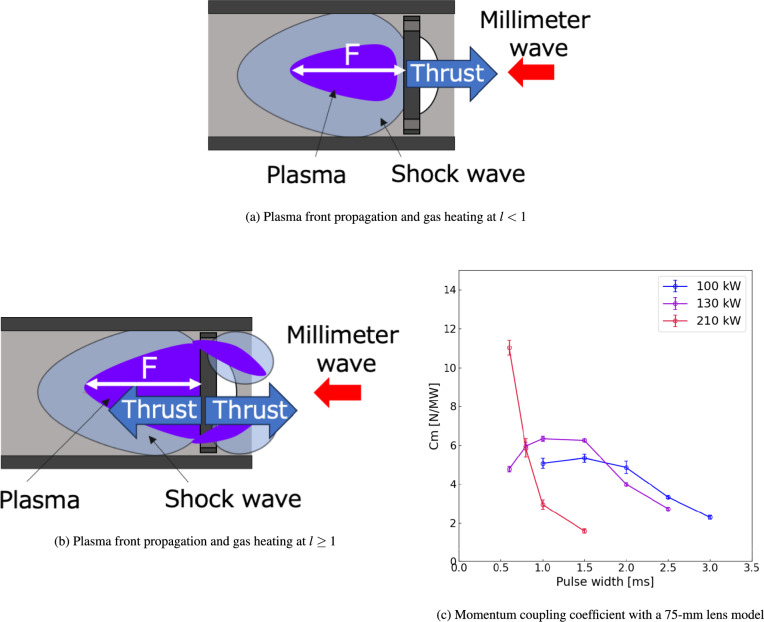
Schematic of plasma-front propagation and momentum coupling coefficients $C_m$ for a lens with a focal length of 75 mm. In the schematics, *l* is the nondimensional length for the plasma-front propagation. The momentum coupling coefficients are evaluated using a pendulum-type impulse measurement device. The beam powers and pulse widths $\tau$ are changed to examine the parameter dependence of propulsive performance.

Because the plasma protrusion from the cylindrical part of the vehicle can decrease the thrust performance, a detailed condition in which this plasma protrusion occurs was examined. Figure 5a and b show geometrical images of the thruster with and without plasma protrusion from the front side. Because the plasma-front propagation distance from the focal point was evaluated as $\tau U_\textrm{i}$ with the pulse width $\tau$ and the time-averaged propagation speed of the plasma front $U_\textrm{i}$, geometrical descriptions for the thruster can predict that plasma protrusion from the front of the vehicle occurs when $\tau U_\textrm{i}$ is greater than the focal length of the lens *F*, as described by the following equation:

12 $$\begin{aligned} l \equiv \frac{\tau U_\textrm{i}}{F} \ge 1, \end{aligned}$$

where *l* is the dimensionless length of plasma front propagation. As indicated in Eq. (12), under the constant beam power, satisfying $l<1$ is important by decreasing $\tau$ to confine the plasma front inside the cylindrical part of the vehicle and reduce the energy loss. In addition to decreasing $\tau$, the plasma-front protrusion to the front of the vehicle can be avoided when the plasma-front propagation speed $U_\textrm{i}$ is decreased. Because a previous study on 28-GHz beam breakdown indicated that a lower $U_\textrm{i}$ is obtained by decreasing the beam power^10^, decreasing the beam power could be effective in satisfying $l<1$. This lower $U_\textrm{i}$ condition was experimentally examined by irradiating the beam with a 130-kW power onto the vehicle, as shown in Fig. 4b, showing that a decrease in the light emission from the front of the vehicle was achieved by decreasing the beam power even if the pulse width $\tau$ of more than 1 ms was selected. This revealed that plasma protrusion was avoided by decreasing *l*. The higher thrust performance could be expected in lower $U_\textrm{i}$ and $\tau$; however, because decreasing the beam power could weaken the shock wave strength induced inside the thruster due to a decrease in the local electric-field intensity and there could be a trade-off relationship between the shock wave strength and the energy loss due to the plasma protrusion, a detailed evaluation for the thrust performance is required for the parameters of the beam power and the pulse width.

### Thrust performance measurement for thruster with a 75-mm focal length lens

To evaluate the thrust performance of the TMiP, the momentum coupling coefficient $C_m$, representing thrust efficiency relative to input beam power, was assessed for a vehicle with a 75-mm focal length lens, as shown in Fig. 5c. The impulse obtained by beam irradiation was measured using a pendulum-type impulse measurement device and a curve that fit the pendulum displacement. A positive $C_m$ was achieved at all parameter ranges of beam power and pulse width, demonstrating that the proposed vehicle could move toward the beam source direction and could be used as a tractor-type beaming propulsion system. Although the plasma protrusion from the front of the vehicle was avoided by satisfying $l \le 1$, at the beam powers of 100 kW and 130 kW, the $C_m$ was small at the pulse widths below 1.0–1.5 ms. This is because the amount of energy input to the vehicle, which was evaluated as $P_b\tau$ ($P_b$ is the beam power), decreased with lower $P_b$, and sufficient gas heating was not obtained, resulting in the induction of the shock wave with insufficient strength. Conversely, the gas heating inside the cylindrical part of the vehicle became stronger with an increase in the pulse width and $P_b\tau$, generating a higher $C_m$. However, the gas heating region at the plasma front escaped from the front of the vehicle when a longer pulse width of $\tau > 2$ ms was used, as described by $l \ge 1$ in Eq. (12). At this more extended pulse width, gas heating and shock wave generation occurred upstream of the front of the vehicle, in addition to the inside of the cylinder of the vehicle, acting on the vehicle as a negative thrust and preventing the vehicle from achieving a high impulse, as shown in Fig. 5a and b. As a result, $C_m$ at 100 kW and 130 kW had a peak value between the pulse widths of 1.0–1.5 ms.

Furthermore, comparing $C_m$ between 100 kW and 130 kW revealed that the maximum $C_m$ at 130 kW exceeded that at 100 kW because the stronger gas heating was obtained due to the higher energy input to the vehicle, as described by $P_b \tau$, inducing the stronger shock wave. At this maximum $C_m$, the plasma protrusion and subsequent energy loss from the front of the vehicle were not enhanced because of the short pulse width of 1 ms, as shown in Fig. 4b. Therefore, at this pulse width, $C_m$ was increased with higher beam power input to the vehicle. However, at the longer pulse width of $\tau \ge 2.0$ ms, $C_m$ at 130 kW decreased rapidly and fell below that at 100 kW. Additionally, the pulse width providing the maximum $C_m$ was shifted to the shorter $\tau$ with an increase in the incident beam power. These characteristics for $C_m$ could be explained by the plasma-front protrusion from the front of the vehicle and the nondimensional length *l*. Because the propagation speed of the plasma front $U_\textrm{i}$ increased with higher beam power, $l \ge 1$ was satisfied even at the shorter pulse width, resulting in the plasma protrusion and a decrease in the thrust performance at the shorter $\tau$. Therefore, at 130 kW, the peak position of $C_m$ shifted to a shorter $\tau$. Conversely, $C_m$ showed no peak at 210 kW and decreased monotonically with increasing pulse width. This monotonic decrease occurred because of an increase in $U_\textrm{i}$; the plasma front protruded from the front of the vehicle even at the pulse width of 0.6 ms, as shown in the plasma image at $\tau =0.6$ ms (Fig. 4a). Although the plasma protrusion occurred, $C_m$ at 210 kW and $\tau =0.6$ ms exceeded those at 100 kW and 130 kW because enhancement of the gas heating and the shock wave can overcome a performance decrease by the plasma protrusion. Therefore, if this strong shock wave generation could be maintained while avoiding plasma front protrusion at 210 kW, a higher thrust performance could be achieved. Based on the dimensionless length, *l*, in Eq. (12), $l < 1$ can be achieved with a higher beam power and a longer pulse width when the focal length of the lens (*F*) is increased, resulting in a larger $C_m$. In the next section, the vehicle lens is replaced with one having a 91-mm focal length to avoid plasma-front protrusion and confine the gas-heating region inside the cylinder of the vehicle for a higher power and a longer period.

### Performance improvement by increasing focal length of a lens

**Fig. 6:**
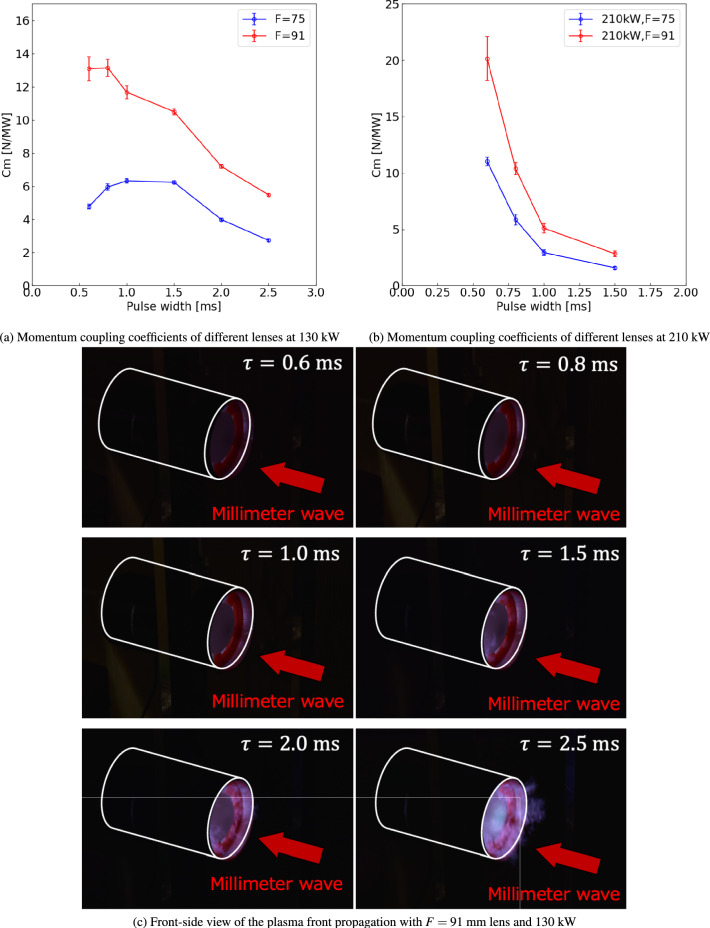
Momentum coupling coefficient $C_m$ and plasma structure with different lenses. The momentum coupling coefficients are evaluated by a pendulum-type impulse measurement device. The hollow observation window is removed from the thruster, and the integral images are captured by a digital camera. The beam powers, pulse widths $\tau$, and focal lengths of the PTFE lens *F* are changed to understand the parameter dependence of propulsive performance and plasma structure.

Figure 6a and b show the momentum coupling coefficients $C_m$ at 130 and 210 kW, respectively, for two PTFE lenses with 75-mm and 91-mm focal lengths mounted on the front of the vehicle. A lens with a longer focal length improved $C_m$ for both 130 and 210 kW. Therefore, as a general conclusion for thruster design, lenses with longer focal lengths should be selected.

At 130 kW and $F=91$ mm, a performance improvement for all pulse widths was achieved, as shown in Fig. 6a. For the longer pulse width of $\tau > 1.5$ ms, as intended, the plasma-front protrusion to the front of the vehicle was suppressed by increasing *F* and decreasing the nondimensional length *l*, reducing the energy loss and improving the thrust performance. This protrusion suppression was confirmed by comparing the pictures in Fig. 6c with those in Fig. 4b, which show that the light emission at the front of the vehicle was weakened. Conversely, for the shorter pulse width of $\tau \le 1.0$ ms, since the plasma protrusion and the sequential performance decrease effect were not strong even at $F=75$ mm, improvement of $C_m$ at $\tau \le 1.0$ ms could not be explained only by suppression of the plasma protrusion. $C_m$ improvement at $\tau \le 1.0$ ms is because the distance between the focal point and the downstream exit of the vehicle (the open-end exit) was decreased from 43 mm to 27 mm by changing $F=75$ mm to $F=91$ mm, shortening a distance between the position where the intense gas heating occurs and the open-end exit. Therefore, at $F=91$ mm, an expansion wave driven by millimeter-wave heating was rapidly exhausted to the open-end exit, inducing a positive pressure wave via open-end reflection of the expansion wave, as indicated in previous computational fluid dynamics (CFD) simulations for a nozzle-type thruster^18^. This pressure wave propagates from the downstream exit to the head of the vehicle, recovering the negative thrust generated by the expansion wave. Therefore, by introducing the longer focal length and shifting the gas heating position to the downstream exit, $C_m$ was improved at the shorter pulse width of $\tau \le 1.0$ ms. Although $C_m$ was enhanced for all pulse widths by the effects mentioned above, a monotonic decrease occurred with an increase in the pulse width. As shown in Fig. 6c, even at a longer focal length of $F=91$ mm, plasma protrusion from the vehicle front could not be avoided at longer pulse widths; therefore, further improvement of the vehicle is required to achieve a higher thrust performance. Attempts to improve thrust performance are discussed in the next section.

At 210 kW, performance improvement was achieved by increasing the focal length to $F=91$ mm, similar to the trend at 130 kW. This is because the plasma front and gas heating area can be confined inside the cylinder of the vehicle for a longer time by decreasing the nondimensional length *l*. The peak of $C_m$ was not generated, and a monotonic decrease in the pulse width was obtained, as with the $F=75$ mm model, meaning that due to the faster propagation speed of the plasma front, complete suppression of the plasma front protrusion was not achieved for all pulse widths, even if the focal length of the lens was increased from 75 mm to 91 mm. Further improvement in the thrust performance could be obtained using a focal length longer than $F=91$ mm; however, an increase in the focal length could increase the total weight of the vehicle, which is disadvantageous for space transportation systems. Therefore, in addition to increasing the focal length, another approach for improving thrust performance by performing the CFD simulation is discussed in the next section.

### Numerical simulation for improving the thrust performance

In this section, a design idea to achieve further improvement in thrust performance is examined by combining a three-dimensional (3D) finite-difference time-domain (FDTD) model, which can reproduce electromagnetic wave propagations, with a 2D axisymmetric CFD simulation.

**Fig. 7:**
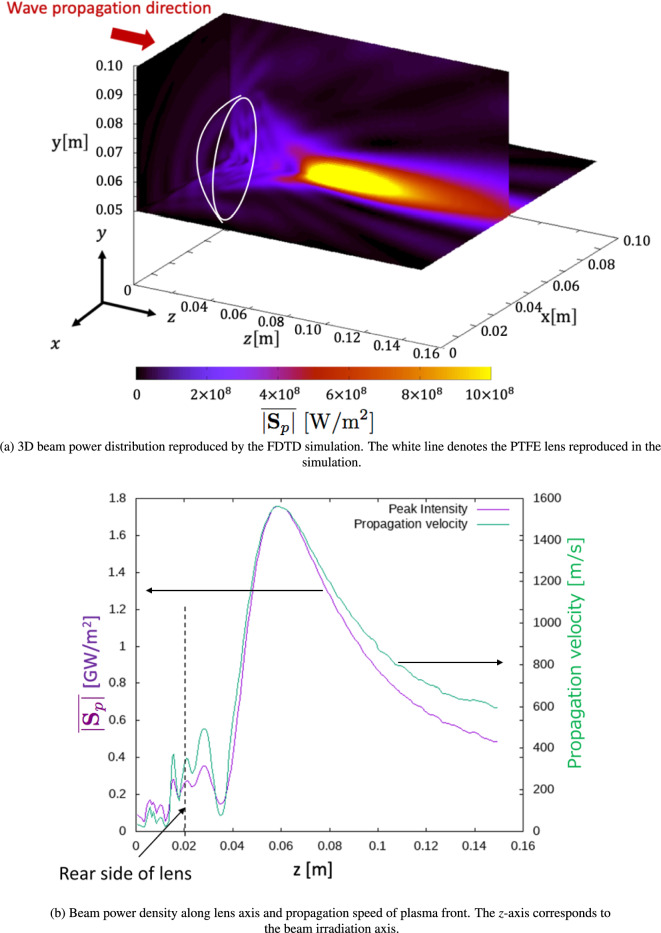
Beam power density calculated by the 3D FDTD simulation and the plasma-front propagation speed estimated by a combination of the FDTD and previous experiment^10^. $\overline{|\textbf{S}_\textrm{p}|}$ denotes the one-period average of the absolute value of the Poynting vector.

Figure 7a shows the beam power density obtained by the 3D FDTD simulation when beams of 210 kW and 28 GHz were irradiated from the boundary at $z=0$ m to the PTFE lens (written as a white line) with $F=75$ mm, indicating that the beam focusing and the high electric intensity region were generated via the PTFE lens. Gas breakdown and plasma generation can be induced by an intense beam intensity around the focal point. Although a high beam intensity was obtained, the position at which the maximum beam intensity was achieved was 40 mm behind the rear surface of the lens, which was shorter than the designed focal length of 75 mm, as shown in Fig. 7b. This difference in the focal length may arise because the lens shape was initially designed for irradiations of 500-GHz beams instead of 28 GHz. Additionally, although the lens was designed to irradiate parallel beams, a Gaussian beam with a divergence angle was used in both the numerical simulation and experimental setup. This decrease in focal length could increase the nondimensional length *l*, which could be a reason for the reduction of thrust performance.

**Fig. 8:**
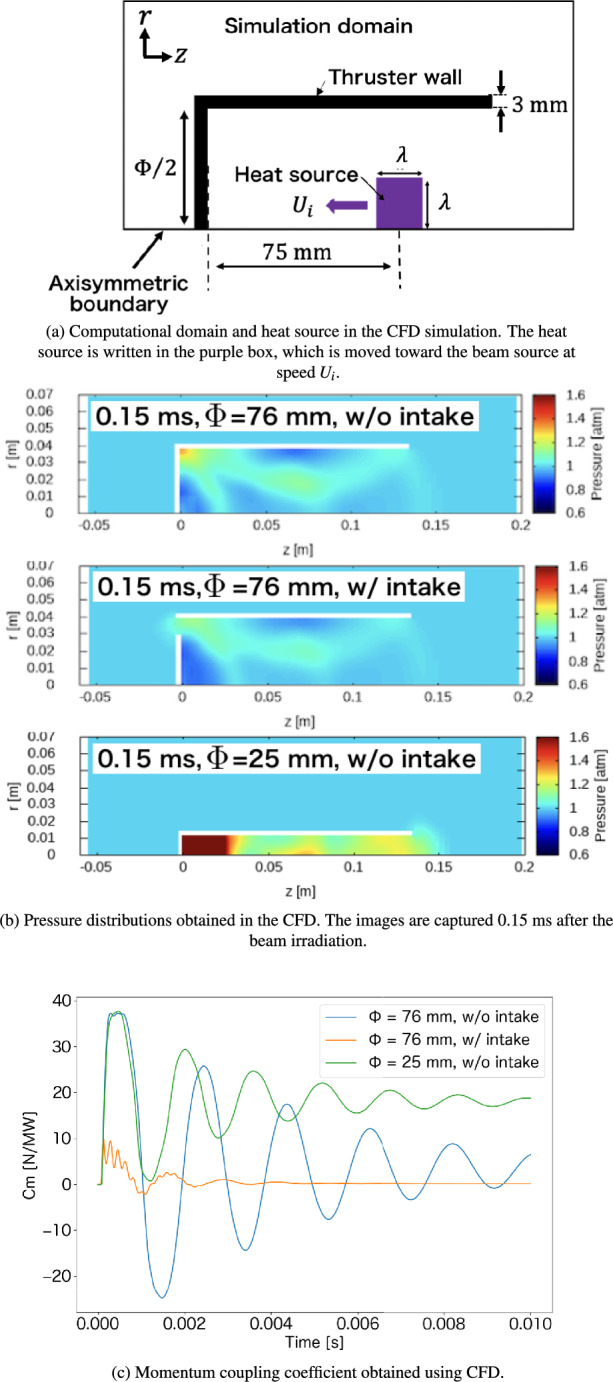
CFD simulation domain. Pressure distributions and momentum coupling coefficient $C_m$ obtained by the CFD simulations. $\Phi$ denotes the thruster diameter.

Using the beam-density profile obtained in the 3D FDTD, the 1D profile of the Poynting vector $\textbf{S}_\textrm{p}$ was calculated along the *z*-axis (Fig. 7b), which was used to model the gas-heating region with *z*-position dependency in the CFD simulation, as indicated in Fig. 8a. Additionally, as shown by the green line in Fig. 7b, the propagation speed of the plasma front ($U_\textrm{i}$) was estimated at each *z*-position based on a linear interpolation between the local Poynting vector obtained by us and the data table experimentally obtained by Tabata et al.^10^. The gas-heating region placed in the simulation domain moved toward the lens mounted on the front of the vehicle at a propagation speed of $U_\textrm{i}$. Gas heating inside the thruster induces a shock wave, interacting with the thruster head (lens) and providing a propulsive force to the thruster, as shown in Fig. 8b. Subsequently, an expansion wave is formed behind the shock wave and interacts with the thruster head, generating a negative thrust. However, the net impulse and $C_m$ were positive for all cases of the different thruster diameter $\Phi$ and the intake design because a positive pressure contribution at an early stage of the shock wave propagation was larger than a contribution of the negative pressure wave, as shown in Fig. 8c. Here, in Fig. 8b and c, the vehicle with a diameter of $\Phi =76$ mm and with the air-breathing intake corresponded to the model used in the above experiment, showing that the simulated $C_m$ was smaller than that obtained in the experiment. This could be because the electromagnetic wave enhancement due to the interaction between the plasma and the incident beam was ignored in the simulation model, and the detailed energy transfer process to the gas heating was ignored. However, a qualitative comparison between each case could be conducted using the simulation.

As shown in Fig. 8b, in $\Phi =76$ mm, comparing the thrusters with and without the air-breathing intake shows that the shock wave was leaked out of the intake mounted on the front of the vehicle, while this was confined inside the thruster without the intake. This outflow of the shock wave can decrease $C_m$. Additionally, in the thruster with air intake, after shock wave propagation, the entire surface of the thruster head interacted with the negative pressure of the expansion wave, decreasing $C_m$. Thus, as indicated in the simulation, because the thruster used in the experiment had an air-breathing intake, $C_m$ was lower than that of the other microwave rocket models. Moreover, without the intake, comparing the thrusters of $\Phi =$ 76 mm and $\Phi =25$ mm revealed that a decrease in the inner diameter of the thruster had a significant effect on an increase in $C_m$. This is because the shock wave is confined inside the narrow cylindrical body of the thruster, enhancing the pressure inside the thruster, as shown in the red area in Fig 8b. In addition to shock wave enhancement, air-breathing from the downstream exit of the vehicle could be enhanced using a narrow cylinder tube, recovering the negative thrust and maintaining a high $C_m$. Therefore, $C_m$ in the experiment was low because the cylindrical part of the vehicle had a larger radius than the amount of energy deposited inside the thruster and could not confine the shock wave driven by gas heating. As a general conclusion for the design criteria of the vehicle, in addition to selecting the appropriate nondimensional length, such as $l < 1$, it is necessary to use a smaller-radius cylinder for the thruster to achieve a larger $C_m$. Additionally, valves were required to cover the air-breathing port, as discussed in previous works^5,18^. These concepts will be examined in future studies.

## Conclusion

This study proposes a tractor millimeter-wave beam propulsion (TMiP) concept driven by a millimeter-wave beam irradiated from the front side of a rocket. Plasma generation and thrust measurement experiments were conducted on a TMiP thruster model. The TMiP consists of a cylindrical main body, an air-breathing port on the front side, a polytetrafluoroethylene (PTFE) lens for beam focusing, and an ignition pin placed around the focal point of the lens. In the experiment, 28-GHz beams with more than 100-kW power were irradiated from the front to rear sides of the vehicle and focused through a PTFE lens mounted on the front side. Camera observations revealed that the high electric-field intensity around the focal point induced gas breakdown and dense plasma propagation toward the lens. Gas heating occurs at the plasma front via energy transfer from the plasma to the neutral gas, causing a strong shock wave inside the cylinder of the vehicle body. The high pressure of the shock wave interacted with the PTFE lens, generating a propulsive thrust that pushed the vehicle toward the beam source direction.

A pendulum-type impulse measurement device was used to measure the momentum coupling coefficient ($C_m$) of the TMiP, which showed that the net impulse became positive. Therefore, the experimental results demonstrate that the TMiP concept is helpful as a propulsion technology. With shorter pulse widths of the incident beam, the gas heating inside the vehicle cylinder was insufficient, weakening the shock wave induction and reducing the thrust. However, when a longer pulse width is used, the plasma front with a strong gas-heating region protrudes from the front of the vehicle, increasing the energy loss and decreasing the thrust performance. Therefore, the plasma-front propagation distance, which can be determined by $U_i\tau$, should be smaller than the focal length of the PTFE lens (*F*), where $U_i$ was the averaged propagation speed of the plasma front and $\tau$ was the pulse width of millimeter-wave beams. This meant that the nondimensional length ($l \equiv (\tau U_\textrm{i})/F$) must be less than unity to obtain the high thrust performance. To decrease the nondimensional length *l* and improve the thrust performance, the focal length *F* was increased from 75 to 91 mm by replacing the PTFE lens on the front of the vehicle. This change improved $C_m$ by suppressing plasma front protrusion, confining the gas-heating region inside the cylinder of the vehicle for a longer duration.

A thruster design concept to improve performance was examined by conducting three-dimensional (3D) finite-difference time-domain (FDTD) and two-dimensional (2D) axisymmetric computational fluid dynamics (CFD) simulations. 3D FDTD simulations of electromagnetic wave propagation evaluated the Poynting vector profile during the PTFE lens focusing process, which was used to model the heating source term in the CFD simulation to drive the shock wave. Shock wave propagation inside the thruster was numerically reproduced, demonstrating that the shock wave leaked out of the air-breathing intake in front of the vehicle. This leakage reduced the surface pressure at the thruster head, thereby decreasing $C_m$. Additionally, the simulation revealed that the thruster radius has a significant effect on thrust performance. The inner diameter of the vehicle cylinder should be smaller than that of the model used in the experiment to confine the shock wave and increase the pressure inside the thruster. In addition, for better thrust performance, the energy conversion efficiency from the incident beam to the impulse can be considered an important factor. In the beaming propulsion concept, the incident beam energy is first converted into plasma energy. Subsequently, plasma energy is transferred into heavy particle energy via relaxation and quenching processes, which is finally converted into impulse. The previous study^33^ indicated that the energy conversion efficiency from the beam to the impulse could be in a range of 10% to 30%, which should be similar to our thruster. However, the exact energy conversion efficiency has not been evaluated from our experimental data; therefore, this will be examined by comparing the results of future experiments with those of the simulations.

In future launch missions, because the power density of the incident microwave beam increases in a realistic rocket, strong electromagnetic waves may affect the onboard electronics and subsystems. Because high-power microwave beams can cause interference, heating, and electronic destruction, they must be shielded with highly reflective shielding devices. When designing and developing such a shielding device, the reflective performance of the shielding material (an aluminum alloy can be the most likely candidate) depends on its surface-processing precision and beam wavelength. Lasers of THz-band beams with shorter wavelengths require high-precision surface processing of the shielding material. However, for GHz-band microwave beams with longer wavelengths, the constraints on the surface processing accuracy of the shielding material are minimal. In addition, the cutoff length for gaps in the shielding devices is orders of magnitude larger for microwave beams than for lasers or THz-band beams, making the geometric design, development, and mass production of shielding devices relatively simple. Therefore, even with increased beam power for practical rocket launch, shielding of subsystems is possible. In addition, because of the large tolerance for dimensional accuracy, shielding devices are relatively inexpensive to develop. These practical shield designs for subsystems will be discussed in future studies.

## Data Availability

Requests for materials or codes should be addressed to Masayuki Takahashi.

## References

[1] Oda, Y. & Komurasaki, K. Plasma generation using high-power millimeter-wave beam and its application for thrust generation. *J. Appl. Phys.* **100**, 113307 (2006).

[2] Oda, Y. et al. Thrust performance of a microwave rocket under repetitive-pulse operation. *J. Propul. Power* **25**, 118–122 (2009).

[3] Oda, Y. et al. A study on the macroscopic self-organized structure of high-power millimeter-wave breakdown plasma. *Plasma Sources Sci. Technol.* **29**, 075010 (2020).

[4] Kakinuma, K. et al. Two-stage-to-orbit transporting system combining microwave rocket and microwave thermal rocket for small satellite launch. *Trans. JSASS Aerospace Tech. Japan* **14**, 99–103 (2016).

[5] Fukunari, M., Arnault, A., Yamaguchi, T. & Komurasaki, K. Replacement of chemical rocket launchers by beamed energy propulsion. *Appl. Optics* **53**, 16–22 (2014). 10.1364/AO.53.000I16 25402933

[6] Nakamura, Y. & Komurasaki, K. Theory and modeling of under-critical millimeter-wave discharge in atmospheric air induced by high-energy excited neutral-particles carried via photons. *Plasma Sources Sci. Technol.* **29**, 105017 (2020).

[7] Vikharev, A. L., Gorbachev, A. M., Kim, A. V. & Kolysko, A. L. Formation of the small-scale structure in a microwave discharge in high-pressure gas. *Soviet J. Plasma Phys.* **18**, 554–560 (1992).

[8] Bogatov, N. A. et al. Gasdynamic propagation of a nonequilibrium microwave discharge. *Soviet J. Plasma Phys.* **12**, 415–420 (1986).

[9] Hidaka, Y. et al. Observation of large arrays of plasma filaments in air breakdown. *Phys. Rev. Lett.* **100**, 035003 (2008). 18232990 10.1103/PhysRevLett.100.035003

[10] Tabata, K. et al. Experimental investigation of ionization front propagating in a 28 GHz gyrotron beam: observation of plasma structure and spectroscopic measurement of gas temperature. *J. Appl. Phys.* **127**, 063301 (2020).

[11] Shimamura, K. et al. Propagation of microwave breakdown in argon induced by a 28 GHz gyrotron beam. *Phys. Plasmas* **28**, 033505 (2021).

[12] Shimamura, K. et al. Wireless power transmission efficiency for microwave rocket using 28 GHz gyrotron. *J. Spacecraft Rockets* **57**, 632–635 (2020).

[13] Takahashi, M. & Ohnishi, N. Computational studies for plasma filamentation by magnetic field in atmospheric microwave discharge. *Appl. Phys. Lett.* **105**, 223504 (2014).

[14] Takahashi, M. & Ohnishi, N. Thrust performance of microwave rocket at low ambient pressure. *Trans. Jpn. Soc. Aeronaut. Space Sci. Aerosp. Technol. Jpn.* **14**, 209–215 (2016).

[15] Takahashi, M. & Ohnishi, N. Plasma filamentation and shock wave enhancement in microwave rockets by combining low-frequency microwaves with external magnetic field. *J. Appl. Phys.* **120**, 063303 (2016).

[16] Takahashi, M. & Ohnishi, N. Numerical study of breakdown pattern induced by an intense microwave under nitrogen and argon gases. *Jpn. J. Appl. Phys.* **55**, 07LD02 (2016).

[17] Takahashi, M., Kageyama, Y. & Ohnishi, N. Joule-heating-supported plasma filamentation and branching during subcritical microwave irradiation. *AIP Adv.* **7**, 055206 (2017).

[18] Takahashi, M. & Ohnishi, N. Open-front approach of a microwave rocket sustained by a resonant magnetic field. *J. Propul. Power* **34**, 762–771 (2018).

[19] Takahashi, M. & Ohnishi, N. Gas propellant dependency of plasma structure and thrust performance of microwave rocket. *J. Appl. Phys.* **125**, 163303 (2019).

[20] Takahashi, M. Coupling simulation on two-dimensional axisymmetric beaming propulsion system. *J. Phys. Conf. Ser.* **2207**, 012047 (2022).

[21] Takahashi, M. & Ohnishi, N. Thrust-performance maximization of microwave rocket sustained by resonant magnetic field. *Trans. Jpn. Soc. Aeronaut. Space Sci. Aerosp. Technol. Jpn.* **17**, 531–537 (2019).

[22] Takahashi, M. & Ohnishi, N. Postural control for beam-riding flight of a microwave rocket using an external magnetic field. *Trans. Jpn. Soc. Aeronaut. Space Sci. Aerospace Technol. Jpn.* **17**, 525–530 (2019).

[23] Takahashi, M. & Ohnishi, N. Gas-species-dependence of microwave plasma propagation under external magnetic field. *J. Appl. Phys.* **124**, 173301 (2018).

[24] Takahashi, M. Development of plasma fluid model for a microwave rocket supported by a magnetic field. *J. Phys. Conf. Ser.* **905**, 012024 (2017).

[25] Takahashi, M. Asymmetric shock wave generation in a microwave rocket using a magnetic field. *J. Phys. Conf. Ser.* **905**, 012020 (2017).

[26] Takahashi, M. Microwave-driven in-tube accelerator. *J. Propul. Power* (under review). 10.1038/s41598-025-08430-3 PMC12234760 40624064

[27] Suzuki, S., Hamasaki, K., Takahashi, M., Kato, C. & Ohnishi, N. Numerical analysis of structural change process in millimeter-wave discharge at subcritical intensity. *Phys. Plasmas* **29**, 093507 (2022).

[28] Suzuki, S., Kato, C., Takahashi, M. & Ohnishi, N. Plasma propagation via radiation transfer in millimeter-wave discharge under subcritical condition. *J. Phys. Conf. Ser.* **2207**, 012046 (2022).

[29] Suzuki, S. & Takahashi, M. Numerical simulation of electromagnetic-wave interference induced by ionization-front of millimeter-wave discharge at subcritical conditions and application to discharge structure identification. *J. Appl. Phys.* **136**, 153301 (2024).

[30] Myrabo, L. N., Messitt, D. G. & Mead, F. B. Jr. Ground and flight tests of a laser propelled vehicle. AIAA Paper 98-1001 (1998).

[31] Mead, F. B., Jr., Messitt, D. G. & Myrabo, L. N. Flight and ground tests of a laser-boosted vehicle. AIAA Paper 98-3735 (1998).

[32] Myrabo, L. N. World record flights of beam-riding rocket lightcraft - demonstration of “disruptive” propulsion technology. AIAA Paper 2001-3798 (2001).

[33] Mori, K., Komurasaki, K. & Arakawa, Y. Energy transfer from a laser pulse to a blast waves in reduced-pressure air atmospheres. *J. Appl. Phys.* **95**, 5979–5983 (2004).

[34] Mori, K. Laser propulsion using a porous carbon heat exchanger. *J. Propul. Power* **38**, 880–883 (2022).

[35] Mori, K. Laser-propelled launch of a spherical capsule guided by a donut-mode beam. *J. Spacecraft Rockets* **54**, 1183–1184 (2017).

[36] Shimamura, K. et al. Internal structure of laser supported detonation waves by two-wavelength Mach-zehnder interferometer. *J. Appl. Phys.* **109**, 084910 (2011).

[37] Sinko, J. E. & Phipps, C. R. Modeling CO laser ablation impulse of polymers in vapor and plasma regimes. *Appl. Phys. Lett.* **95**, 131105 (2009).

[38] Libeau, M. & Myrabo, L. N. Off-axis and angular impulse measurements on a lightcraft engine. In *Proceedings of Third International Symposium on Beamed Energy Propulsion* vol. 766, 166–177 (2005).

[39] Kenoyer, D. A., Anderson, K. S. & Myrabo, L. N. Calibration and validation of a 6-DOF laser propelled lightcraft flight dynamics model vs. experimental data. In *Proceedings of Fifth International Symposium on Beamed Energy Propulsion* vol. 997, 325–337 (2008).

[40] Ballard, C. G., Anderson, K. S. & Myrabo, L. N. Flight dynamics and simulation of laser propelled lightcraft. *J. Comput. Nonlinear Dyn.* **4**, 041005 (2009).

[41] Scharring, S., Eckel, H. A. & Röser, H. P. Beam-riding analysis of a parabolic laser-thermal thruster. In *Proceedings of Seventh International Symposium on Beamed Energy Propulsion* vol. 1402, 115–131 (2011).

[42] Scharring, S., Eckel, H. A. & Röser, H. P. Beam-riding of a parabolic laser lightcraft. *Int’l J. Aerosp. Innov.* **3**, 15–31 (2011).

[43] Scharring, S., Hoffmann, D., Eckel, H. A. & Röser, H. P. Stabilization and steering of a parabolic laser thermal thruster with an ignition device. *Acta Astronautica* **65**, 1599–1615 (2009).

[44] Sasoh, A. Laser-driven in-tube accelerator. *Rev. Sci. Instrum.* **72**, 1893 (2001).

[45] Katsurayama, H., Komurasaki, K. & Arakawa, Y. Numerical analyses on pressure wave propagation in repetitive pulse laser propulsion. AIAA Paper 2001-3665 (2001).

[46] Katsurayama, H., Komurasaki, K. & Arakawa, Y. Computational performance estimation of laser ramjet vehicle. AIAA Paper 2002-3778 (2002).

[47] Takahashi, M. & Ohnishi, N. Beam riding performance of asymmetrically propelled laser vehicle. *AIAA J.* **50**, 2600–2608 (2011).

[48] Takahashi, M. & Ohnishi, N. Beam-riding flight of a laser propulsion vehicle using actively controlled pulse. *J. Propul. Power* **32**, 237–250 (2016).

[49] Takahashi, M. & Ohnishi, N. Theoretical and numerical studies of dynamic scaling of a six-degree-of-freedom laser propulsion vehicle. *Int’l J. Aerosp. Eng.* **2015**, 801371 (2015).

[50] Krupka, J. Measurements of the Complex Permittivity of Low Loss Polymers at Frequency Range from 5 GHz to 50 GHz. *IEEE Microw. Wirel. Compon. Lett.* **26**, 464–466 (2016).

[51] Mur, G. Absorbing boundary condition for the finite-difference approximation of the time-domain electromagnetic-field equations. *IEEE Trans. Electromagn. Compat.* **EMC–23**, 377–382 (1981).

[52] Wada, Y. & Liou, M. S. A flux splitting scheme with high-resolution and robustness for discontinuous, AIAA Paper 94-0083 (1994).

[53] Leer, B. V. Toward the ultimate conservative difference scheme V, a second-order sequel to Godunov’s method. *J. Comput. Phys.* **32**, 101–136 (1979).

[54] Kourtzanidis, K., Rogier, F. & Boeuf, J. P. Gas heating effects on the formation and propagation of a microwave streamer in air. *J. Appl. Phys.* **118**, 103301 (2015).

